# Experimental kernel-based quantum machine learning in finite feature space

**DOI:** 10.1038/s41598-020-68911-5

**Published:** 2020-07-23

**Authors:** Karol Bartkiewicz, Clemens Gneiting, Antonín Černoch, Kateřina Jiráková, Karel Lemr, Franco Nori

**Affiliations:** 1 Faculty of Physics, Adam Mickiewicz University, 61-614 Poznan, Poland; 2 RCPTM, Joint Laboratory of Optics of Palacký University and Institute of Physics of Czech Academy of Sciences, 17. listopadu 12, 771 46 Olomouc, Czech Republic; 3 Theoretical Quantum Physics Laboratory, RIKEN Cluster for Pioneering Research, Wako-shi, 351-0198 Japan; 4 Department of Physics, The University of Michigan, Ann Arbor, MI 48109-1040 USA

**Keywords:** Quantum information, Qubits, Quantum optics

## Abstract

We implement an all-optical setup demonstrating kernel-based quantum machine learning for two-dimensional classification problems. In this hybrid approach, kernel evaluations are outsourced to projective measurements on suitably designed quantum states encoding the training data, while the model training is processed on a classical computer. Our two-photon proposal encodes data points in a discrete, eight-dimensional feature Hilbert space. In order to maximize the application range of the deployable kernels, we optimize feature maps towards the resulting kernels’ ability to separate points, i.e., their “*resolution*,” under the constraint of finite, fixed Hilbert space dimension. Implementing these kernels, our setup delivers viable decision boundaries for standard nonlinear supervised classification tasks in feature space. We demonstrate such kernel-based quantum machine learning using specialized multiphoton quantum optical circuits. The deployed kernel exhibits exponentially better scaling in the required number of qubits than a direct generalization of kernels described in the literature.

## Introduction

Many contemporary computational problems (like drug design, traffic control, logistics, automatic driving, stock market analysis, automatic medical examination, material engineering, and others) routinely require optimization over huge amounts of data^1^. While these highly demanding problems can often be approached by suitable *machine learning* (ML) algorithms, in many relevant cases the underlying calculations would last prohibitively long. Quantum ML (QML) comes with the promise to run these computations more efficiently (in some cases exponentially faster) by complementing ML algorithms with quantum resources. The resulting speed-up can then be associated with the collective processing of quantum information mediated by quantum entanglement.

There are various approaches to QML, including linear algebra solvers, sampling, quantum optimization, or the use of quantum circuits as trainable models for inference (see, e.g., Refs.^2–18^). A strong focus in QML has been on deep learning and neural networks. Independently, *kernel-based* approaches to supervised QML, where computational kernel evaluations are replaced by suitable quantum measurements, have recently been proposed^10,12^ as interesting alternatives. Combining classical and quantum computations, they add to the family of quantum-classical hybrid algorithms.

*Kernel-based* QML (KQML)is particularly attractive to be implemented on linear-optics platforms, as quantum memories are not required. Here, we thus investigate the prospect of KQML with multiphoton quantum optical circuits. To this end, we propose kernels that scale exponentially better in the number of required qubits than a direct generalization of kernels previously discussed in the literature^12^. We also realize this scheme in a proof-of-principle experiment demonstrating its suitability on the platform of linear optics, thus, proving its practical applicability with current state of quantum technologies.

Let us explain KQML by first recalling some definitions and theorems, and then we overview the recently proposed method for finding linear boundaries in feature Hilbert space (FHS)^12^. FHS is defined as a space of complex vectors $|\varphi (x)\rangle ,$ where $\varphi$ describes a feature map (FM), and *x* denotes a real vector of dimension *D* (the input data). FHSs generally have higher dimension than the original data *x*. This implies that linear decision boundaries in FHS can give rise to nonlinear decision boundaries in the original data space. By virtue of such nonlinear FMs, it is not required to implement nonlinear transformations on the quantum-state encoded data, in contrast to the direct amplitude encoding common in other QML approaches.

The central idea underlying KQML is that inner products of vectors that are mapped into FHS can be directly accessed by measurements, which then suggests to identify these inner products with kernel functions. By physically measuring the kernel functions $\kappa (x',x)=|\langle \varphi (x')|\varphi (x)\rangle |^2$, it is thus possible to bypass their *per pedes* computation on a classical machine. Such measurement-based implementation may, in some cases, be significantly faster than the latter option.

It follows from the representer theorem that a function of the reproducing kernel that minimizes the cost function (a solution to the ML problem) can be written as $f^*(x)=\sum _{m=1}^M a_m \kappa (x,x^m),$ where *M* is the number of training samples, the coefficients $a_m$ are real parameters subject to the training, and *x* belongs to feature space. For a given kernel $\kappa$, the parameters $a_m$ can be found efficiently. The objective of ML is to deliver a function $f^*(x)$ that classifies the non-separable points ${x^1,...,x^{M-K}}$ and ${x^{M-K+1},...,x^M}$ by finding a trade-off between the number of misclassifications and the width of the separating margin. The parameters $a_m$ can be obtained by solving the following problem: minimize $\sum _{m=1}^M (|a_m|^2 + \gamma u_m)$ such that $a_i \kappa (x,x^i) \ge 1 - u_i$ for $i = 1,...,M-K,$ and $a_i \kappa (x,x^i) \le -(1 - u_i)$ for $i = M-K+1,...,M,$ $u \ge 0,$ where $\gamma$ gives the relative weight of the number of misclassified points compared to the width of the margin. In a nutshell, this approach allows to *replace the nonlinearity of the problem with linear multidimensional quantum computations, which offers a potential speed-up.*

## Results

### Kernel resolution in finite dimensions

An important and widespread kernel class are Gaussian-type kernels, which introduce a flexible notion of proximity among data points. An essential hyperparameter of Gaussian-type kernels is thus their variance (or, more generally, their *resolution*). The resolution determines a Gaussian kernel’s ability to distinguish data points, which, for given training data, can decide if a model can be trained successfully or not. If kernel resolution is too coarse, resulting decision boundaries miss relevant details in the data; if it is too refined, the model becomes prone to overfitting. Only if the resolution can be chosen sufficiently flexibly to be accommodated to the structure of the data, model training can be expected to be successful.

In the infinite-dimensional feature spaces offered by continuous variable implementations, viable FMs with (in principle) arbitrary resolution can be implemented, e.g., by mapping data into squeezed states^12^, where the adjustable squeezing factor then determines the resolution of the resulting Gaussian kernel (i.e., its variance). However, within the paradigm of discrete, finite-dimensional quantum information processing, the FHS dimension becomes a scarce resource, resulting in limitations on kernel resolution. As we show now, optimizing the range of kernel resolutions in finite dimensions then forces us to move beyond the scope of Gaussian kernels.

Let us discuss the optimal kernel resolution that can be achieved in *N*-dimensional FHS, within the class of FMs of the form

1 $$\begin{aligned} x \rightarrow |\psi (x)\rangle = \sum _{n=0}^{N} \sqrt{r_n} e^{2 \pi i n x} \, |n\rangle, \quad \sum _{n=0}^{N} r_n = 1, \end{aligned}$$

with $\{|n \rangle \}$ a basis of the Hilbert space and $x \in [-1/2, 1/2)$. Any data set can be brought to this form, which is a routine step in data preparation. We stress that the amplitudes $r_n$ are independent from the input values *x*. The resulting kernels then are of the form

2 $$\begin{aligned} \kappa (x,x') = \kappa (x-x') = \left| {\sum _{n=0}^{N} r_n \, e^{2 \pi i n (x'-x)}}\right| ^2. \end{aligned}$$

In this shorthand notation $\kappa (x) \ge 0 \quad \forall x$ and $\kappa (0)=1$. For the sake of clarity we consider here 1D input data *x*. For *D*-dimensional inputs $\mathbf {x}$, each input component $x_i$ is encoded separately, requiring an $(N\cdot D+D)$-dimensional FHS. If the FHS is spanned by *q* qubits, we have $N=2^q-1$. In particular, for $N=1$ and $r_n = 1/2$ we have $\kappa (x,x')=\cos [\pi (x'-x)]^2,$ which realizes a *cosine kernel* (CK). The class of states (1) comprises also truncated squeezed states $|\psi _{\mathrm{TSQ}}(x)\rangle$, with

3 $$\begin{aligned} \sqrt{r_n} = \frac{\sqrt{(2 n)!} (-\tanh \zeta )^n}{\sqrt{B} \, 2^n n! \sqrt{\cosh \zeta }} \end{aligned}$$

($\zeta$ denotes the squeezing factor and *B* renormalizes the state after truncation), and, what we call here, *multi-slit interference states* $|\psi _{\mathrm{MSI}}(x)\rangle$, with constant amplitudes $\sqrt{r_n} = 1/\sqrt{N}$. The latter inherit their name from the fact that, by virtue of $\langle x|p \rangle = e^{2 \pi i p x}$ (*h*=1), they are formally equivalent to a balanced superposition of momentum states in a (hypothetical) compact continuous variable Hilbert space (augmented by an internal spin-*N* degree of freedom),

4 $$\begin{aligned} |\psi _{\mathrm{MSI}}(x)\rangle = \frac{1}{\sqrt{N}} \sum _{n=1}^{N} \langle x| p=n \rangle |n\rangle , \end{aligned}$$

giving rise to “*N*-slit interference” in the position coordinate when projected onto $\langle x| \otimes \frac{1}{\sqrt{N}} \sum _{n=1}^{N} \langle n|$^19^. Note that polynomial kernels (discussed, e.g., in^8,12^) fall outside of the state class (1).

We can use the above compact-space embedding to gain further insight into the nature of our kernel definition (2). If we interpret the states (1) as $|\psi \rangle = \sum _{n=1}^{N} \sqrt{r_n} |p=n\rangle \otimes |n\rangle$, we can introduce the density operator $\rho = |\psi \rangle \langle \psi |$ and trace over the internal spin degree of freedom,

5 $$\begin{aligned} \rho _{\mathrm{ext}} = {\mathrm{Tr}}_{\mathrm{int}} (\rho) = \sum _{n=1}^{N} r_n |p=n\rangle \langle p=n|. \end{aligned}$$

We then find that the kernel (2) is related to the spatial coherences of the mixed reduced state $\rho _{\mathrm{ext}}$: $\kappa (x,x') = |\langle x|\rho _{\mathrm{ext}}|x' \rangle |^2$.

We define a kernel’s spatial resolution $\Delta x[\kappa ]$ by its variance (a hyperparameter typically optimized for Gaussian kernels)

6 $$\begin{aligned} (\Delta x[\kappa ])^2 \equiv \int _{-1/2}^{1/2} dx \, x^2 {\tilde{\kappa }}(x) , \end{aligned}$$

where the renormalized kernel ${\tilde{\kappa }}(x) = \kappa (x)/R$, with $R \equiv \int _{-1/2}^{1/2} dx \, \kappa (x) = \sum _{n=1}^{N} r_n^2$, describes a valid probability distribution. In the case of the mulit-slit interference states $|\psi _{\mathrm{MSI}}\rangle$, one analytically obtains $(\Delta x[\kappa _{\mathrm{MSI}}])^2 = \frac{1}{12} (1-S_1(N))$, with the interferometric “squeezing factor” $S_1(N) = -\frac{12}{\pi ^2} \sum _{j=1}^{N-1} (-1)^j \frac{N-j}{N j^2}$, and $N \ge 2$^19^.

**Figure 1:**
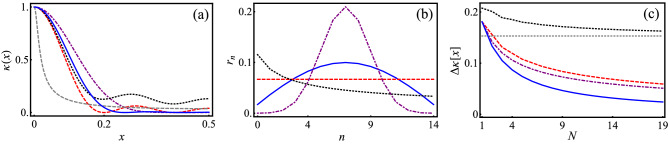
Kernel family (2) for different amplitude choices. (**a**) We find that the resolution-optimized kernel (blue solid) exhibits suppressed side maxima as compared to the MSI kernel (red dashed), while the TSQ kernel (with squeezing factor $\zeta =2$, black dotted) maintains a nonvanishing plateau at all *x* values. For comparison, we also display the respective squeezed-state kernel for $N \rightarrow \infty$ (gray dotted) and CK (purple dash-dotted). (**b**) Characteristic amplitude progressions for the example of $N=14$ and $\zeta =4$. (**c**) The optimized kernel exhibits a significantly improved resolution progression with *N*,  as compared to the MSI or the TSQ kernel (here with $\zeta =3$).

The kernel (6) minimizing the variance is a solution to the optimization problem: minimize $\frac{\mathbf {r}^T \cdot K \cdot \mathbf {r}}{|\mathbf {r}|^2}$ such that $\sum _{n=1}^{N} r_n = 1$, where

7 $$\begin{aligned} K_{n m} = \left\{ \begin{array}{ll} \frac{1}{12}, &{} n=m \\ \frac{(-1)^{|n-m|}}{2 (n-m)^2 \pi ^2}, &{} \, \text {else} \\ \end{array} \right. \end{aligned}$$

and $\mathbf {r}=(r_1, \dots , r_N)^T$. In Fig. 1 we compare this optimized kernel with the TSQ and the MSI kernel. The optimized kernel comes with strongly suppressed side maxima as compared to the MSI kernel, while the TSQ maintains a nonvanishing plateau for all *x* values. Consequently, the optimized kernel enables, for a given *N*, a significantly improved resolution as compared to the other kernel choices. Figure 1b clarifies that amplitudes decaying symmetrically about the “center” state are responsible for improving the kernel resolution.

On the other hand, a kernel that maximizes the variance (i.e., $\kappa (x)=1$) follows from $r_1=1$ and $r_n=0$ for $n\ne 1$, resulting in the variance $(\Delta x[\kappa ])^2=1/12.$ By a suitable choice of the coefficients $r_n$, we can thus tune the resolution of the kernel between its minimum value obtained for the optimized kernel and its maximum value assumed for a uniform kernel.

Whereas kernels of the form (2) can also be efficiently computed classically, their quantum evaluation may still deliver a significant speed-up. We illustrate this with an example, the computation of $\cos ^{2N} x.$ The optimal classical algorithm depends on the properties of *N*. In the best case scenario, *N* is a power of 2. Then, in the first step we compute $\cos ^2 x.$ Next, we compute $[\cos ^2 x]^2,$ etc. The entire computation then takes $\log _2(N+1)$ steps. As we demonstrate below, for the quantum implementation, the required size of the FHS (number of qubits) grows also like $\log _2(N+1)$. However, in contrast, there the associated calculations are replaced by a single measurement. We expect similar arguments to hold for more general classes of functions, as well.

Beyond the quantum-classical hybrid approach pursued here, the proposed FMs may, if seen as modules to be combined with other quantum computing subroutines, contribute their resource-efficient data point separation ability to an overall setup that comes with an inherently quantum scaling advantage. MSI states, for instance, can be generated in a gate-based quantum computer following the first stage of the phase-estimation algorithm^20^.

### Alternative Gaussian-kernel implementation

**Figure 2:**
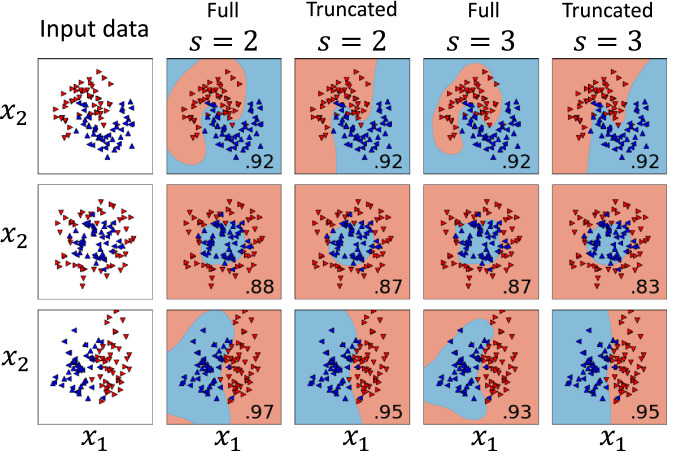
Training results on a random inseparable data set of 40 samples (up/down-tipped triangles). The performance on a test set (left/right-tipped triangles) of 60 points (the fraction of correctly classified samples that were not used in the QML process) is given in the bottom right corner of each respective subplot. We find that the optimal variance/resolution choice for the Gaussian kernel is $s=2$. For $s=3$ we deal with overfitting. Shown are the simulation results both for an exact Gaussian kernel and for the truncated FM (8) comprising 4 terms ($q=2$). The learned classification boundaries are given as contour plots. The slight difference in performance compared to the theoretical prediction is due to statistical fluctuations in the experimental data and the relatively small test set (misclassification of a single near-boundary point results in a 0.02 performance drop).

Above we have shown that truncated squeezed states and their resulting kernels fall within the state class (1). If we relax the condition that the amplitudes $r_n$ be independent from the input values *x*, we can formulate an alternative data encoding into truncated squeezed states according to

8 $$\begin{aligned} |\phi (x)\rangle = Z\sum _{n=0}^{N}\frac{(sx)^n}{\sqrt{n!}}|n\rangle , \end{aligned}$$

where $N=2^q-1$, $x=x_1+ix_2,$ and $Z^{-1}=\sum _{n=0}^{N}\frac{(s|x|)^{2n}}{n!}.$ Note that this feature map is defined for 2D inputs $x=(x_1,x_2)^T$. For large *N* this kernel again reproduces to good approximation a Gaussian kernel, as

9 $$\begin{aligned} \kappa (x',x)=|\langle \varphi (x')|\varphi (x)\rangle |^2\approx \exp{[-s^2(x_1-x'_1)^2-s^2(x_2-x'_2)^2]}, \end{aligned}$$

where the variance is set by the hyperparameter *s*. In particular, as shown in Fig. 2, this approximation is valid for $q=2$ and relatively small values of *s*.

We find that this kernel performs, for the number of qubits $q=2$ and after numerically optimizing the hyperparameter to $s=2$, on average as well as the cosine kernel for the same total number of qubits equal to $N=1$ (see Fig. 4). Moreover, by appropriate parameter reconfiguration, it would be possible to realize this type of kernel using the same experimental setup. From an experimental perspective, however, it is more convenient (and thus scalable) to implement the feature map associated with the powers of the cosine kernel, which is exclusively implemented by setting phases and polarization angles.

### Cosine kernels

The kernel selected for our proof-of-principle demonstration of KQML is

10 $$\begin{aligned} \kappa (x',x)=|\langle \varphi (x')|\varphi (x)\rangle |^2=\prod _{n=1}^{D} \cos ^{2N}(x_n'-x_n), \end{aligned}$$

where the FM taking a normalized feature $x_n\in [-\pi /2,\pi /2)$ to FHS is $|\varphi (x)\rangle =\bigotimes _{n=1}^{D} \sum _{k=0}^{N} \sqrt{N\atopwithdelims ()k} \sin ^k(x_n)\cos ^{N-k}(x_n)|k\rangle _n.$ Note that *N* is related to the number of qubits *q* per dimension as $q=\lceil \log _2(N+1)\rceil .$ This FM can also be considered a constant-phase representation of constant-amplitude states. This is the same as representing states either in a basis of eigenstates of *x* or *z* components of a collective spin operator. In particular, $(\cos (x)|0\rangle + \sin (x)|1\rangle )/\sqrt{2}\Leftrightarrow (|0'\rangle + e^{2ix}|1'\rangle )/\sqrt{2},$ where $|0\rangle = (|0'\rangle +|1'\rangle )/\sqrt{2}$ and $|1\rangle = (|0'\rangle -|1'\rangle )/\sqrt{2}.$

This mapping uses exponentially less resources (qubits) than the direct product of the map from Ref.^12^, i.e., $|\varphi (x)\rangle =\bigotimes _{n=1}^{D} \bigotimes _{m=1}^{N} \sum _{k=0}^1\sin ^k(x_n)\cos ^{1-k}(x_n)|k\rangle _{n,m},$ where the number of qubits per dimension is $q=N.$ Using the powers of CKs allows us to adjust the kernel resolution by choosing the proper value of *N*. Thus, the number of used qubits can be related directly to the variance of the kernel. The number of qubits here plays the same role as the squeezing parameter in the experimental proposal given in Ref.^12^. The CK can also include additional $(D-1)$ degrees of freedom by virtue of a FM defined as

11 $$\begin{aligned} |\varphi (x)\rangle =\bigotimes _{n=1}^{D} \sum _{k=0}^{N}e^{i2y_{n-1}} \sqrt{N\atopwithdelims ()k}\sin ^k(x_n)\cos ^{N-k}(x_n)|k\rangle _n, \end{aligned}$$

where $y_0=0,$ the number of terms here is $(N+1)^D,$ and the associated kernel measured by postselection is $\kappa (x',x)=\prod _{n=1}^D\cos ^{2N}(x'_n-x_n)\cos ^2(y'_{n-1}-y_{n-1}).$

## Discussion

**Figure 3:**
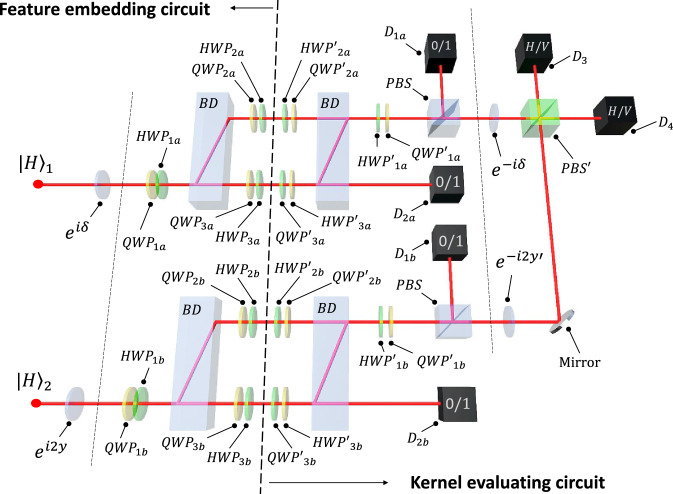
Optical circuit implementing both the FM and the model circuits. The performance of the setup in QML is shown in Fig. 4 for $N=1$ and $D=2.$ The experimental setup consists of polarizing beam splitters (*PBS*s), beam dividers (*BD*s), quarter-wave and half-wave plates (*QWP*s and *HWP*s, respectively), and single photon detectors $D_{n}$ for $n=1a,1b,2a,2b,3,4$. $D_3$ and $D_4$ are *H*/*V* polarization resolving (implemented as a *PBS* and two standard detectors). The kernel $\kappa (x',x)_{\mathrm {exp}} =[\sum _{p,s=H,V} CC(D_{2s},D_{3p}) - CC(D_{2V},D_{3H})+CC(D_{2H},D_{3V})]/ \sum _{m>n}\sum _{n=1}^6 CC(D_{m},D_{n})$ is given as a ratio of coincidences $CC(D_{m},D_{n})$ registered by photon detectors $D_{n}$ and $D_{m}$ to the total number of photons.

**Figure 4:**
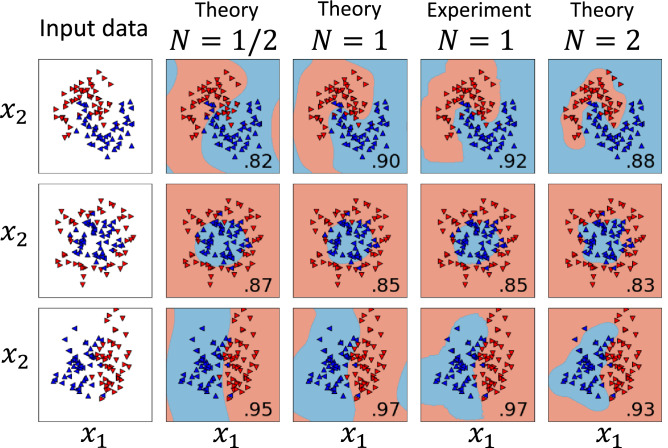
Training results on a random inseparable data set of 40 samples (up/down-tipped triangles). The performance on a test set (left/right-tipped triangles) of 60 points (the fraction of correctly classified samples that were not used in the QML process) is given in the bottom right corner of each respective subplot. We see that the best choice of CK is $N=1$. For $N=2$ we deal with overfitting and for $N=1/2$ the kernel is too coarse to give as good results as for $N=1$. The learned classification boundaries are given as contour plots. The slight difference in performance of KQML in relation to the theoretical prediction is due to statistical fluctuations of the experimental data and relatively small test set (misclassification of a single near-boundary point results in 0.02 performance drop).

We have experimentally implemented KQML to solve three classification problems on a two-photon optical quantum computer. In our experiment we implemented a $D=2,N=1$ kernel (using all the modes from Fig. 3, we can set at most $D=5$ with $q=1$). We used two photons, but only the top mode of the dual-rail encoding. Including more modes would lead to kernels causing overfitting (see Fig. 4).

We have performed measurements for $M=40$ two-dimensional samples ($D=2$), drawn from two classes (see horizontally/vertically-tipped triangles in Fig. 4). This procedure was repeated for three benchmark classification problems. For each benchmark $40\times 39/2=780$ measurements were performed to create a corresponding Gram matrix (GM), which was subsequently used to find the best linear classification boundary as given by the representer theorem. In other words, a custom kernel $\kappa (x^m,x^{n})=\kappa (x^n,x^{m})$ for $m,n=1,2,...,M$ was measured. This kernel was used as a custom precomputed kernel for the scikit-learn SVC classifier in python.

Pairs of *H*-polarized photons were prepared in a type-I spontaneous parametric down-conversion process in a $\beta$–BaB${}_2$O${}_4$ crystal. The crystal was pumped by a 200 mW laser beam at 355 nm (repetition rate of 120 MHz). The coincidence rate, including all possible detection events from Fig. 3, was approximately 250 counts per second. The setup operates with high fidelity (98%) and the dominant source of errors can be attributed the Poissonian photon count statistics. The design of this setup is modular and its easy to incorporate more qubits by simply adding additional blocks. We measured each point for a time necessary to collect about 2,500 detection events. Thus, excluding the time needed to switch the setup parameters, the whole measurement for a single benchmark problem takes about two hours.

To prepare the contour plot of the decision function based on the experimental data shown in Fig. 4 and to quantify the performance of the trained model on the relevant test sets, we have also measured the GM for 1,225 points and used its symmetries to fill in the unmeasured values. The values for points in between have been found using linear interpolation. The data accumulation time can be shortened by orders of magnitudes by fine tuning the parameters of the setup and by using brighter photon sources.

## Conclusions

We report on the first experimental implementation of supervised QML for solving a nonlinear multidimensional classification problem with clusters of points which are not trivially separated in the feature space. We hope that our research on QML will help to improve ML technologies, which are a major power-horse of many industries, a vivid field of research in computer science, and an important technique for solving real-world problems. We believe that both the theoretical and the experimental investigation of FM circuits and their constraints regarding kernel resolution and compression for a limited FHS (i.e., FHS size dependent FMs) constitutes a crucial step in the development of practical KQML for support-vector-machine QML^8–10,12,13^.

We demonstrate that a linear-optical setup with discrete photon encoding is a reliable instrument for this class of quantum machine learning tasks. We also report obtaining exponentially better scaling of FHS in the case of CK than in the case of taking direct products of qubits^12^. The same can hold for other more complex kernels implemented in finite FHS, which could appear unfeasible, but in fact require nontrivial FMs (e.g., the resolution-optimized kernels shown in Fig. 1). Thus, KQML can provide a promising perspective for utilizing noisy intermediate-scale quantum systems^21–24^, complementing artificial quantum neural networks^25–29^ and other hybrid quantum-classical algorithms^30–32^.

The classical computational cost of the power kernel computation is ${\mathcal {O}}[\mathrm {log}(N)]$ and the quantum cost is a constant value depending on the precision of the computation. In the classical case, one needs to perform ${\mathcal {O}}[\mathrm {log}(N)]$ computation steps that can not run in parallel due to the recursive nature of the classical algorithm. In the quantum case, one needs to run 1 computation step but on $\mathrm {log}(N)$ qubits. As in any quantum computation, the precision of the calculation depends on the number of measurements and it can be considered constant for a given computational problem. This observation itself is a valuable result and a quantum advantage. The quantum advantage of the presented approach is apparent in terms of the complexity of calculations, i.e., ${\mathcal {O}}[\mathrm {log}(N)]$ versus ${\mathcal {O}}(1)$. Consider the number of samples needed for quantum calculations. It depends on the confidence level (*z*) and admissible error: $\epsilon$. For a given pair of *z* and error $\epsilon$, one needs ${\mathcal {O}}(1/\epsilon ^2)$ repetitions of the experiment. This is just a constant overhead. In the classical case, this constant overhead can be smaller, but the complexity of calculations can be larger as the it is *N*-dependent. Only if we face significantly lower than unity qubit-number-dependent efficiency $\eta$ (i.e., circuit-size dependent losses), for a given *z*-value the complexity of quantum computations should be considered as being ${\mathcal {O}}(\eta ^(-\mathrm {log}(N))/\epsilon ^2)$. However, the power scaling also applies to the total error probability of classical computations of $\mathrm {log}(N)$ steps. Note, however, that both $\eta$ and single-step error probability of classical computing are not fundamentally limited and can be arbitrary close to 1 or 0, respectively.

Our quantum kernels can be used for solving high-dimensional classification problems and could potentially be computed faster than their classical counterparts. Popular problems solved by classification algorithms include image recognition (e.g. face detection or character recognition), speech recognition (e.g. voice user interfaces), medical diagnoses (e.g. associating results of medical tests with a class of diseases), real-time specific data extraction from vast amounts of unstructured data (e.g. classification of patterns in unstructured data) and many more. Classification can also be used as an initial phase for predictive computations that help to make the best decision based on the available data (e.g., managing risk, security, traffic, procurement etc.). We believe that this quantum-enhanced approach is useful especially in cases where it is difficult or impossible to achieve the result on time with classical computing.

## Methods

### Optical circuit for KQML

States given by Eq. (11) can be prepared in a quantum optical setup. In the reported proof of principle experiment, we can set $N=3$ and $D=2$. This means that, effectively, the experiment deploys $q=2$ qubits per dimension. The FM is defined via single-photon polarization states (*H*/*V* polarization) as well as dual-rail encoding (*T*/*B* for top/bottom rail, respectively)

12 $$\begin{aligned} |\varphi (x)\rangle= & {} \bigotimes _{n=1}^2 \left( c^3(x_n)|HT\rangle _n + \sqrt{3}s(x_n) c^2(x_n)|HB\rangle _n\right. \nonumber \\&\left. + \sqrt{3}c(x_n) s^2 (x_n)|VB\rangle _n + s^3(x_n)|VT\rangle _n \right) , \end{aligned}$$

where $c(x_n)\equiv \cos (x_n)$ and $s(x_n)\equiv \sin (x_n).$ This approach is resource-efficient as it only requires two photons to encode *x* into the FHS state of $N=3$ and $D=2$.

An optical circuit implementing this FM is depicted in Fig. 3. The top part of the FM circuit works as follows: first, it transforms the standard input $|HB\rangle$ using wave plates resulting in $|HB\rangle \rightarrow (|HB\rangle + |VB\rangle )/\sqrt{2}.$ Next, a beam divider separates polarization modes in space, i.e., we have $(|HB\rangle + |VT\rangle ).$ Now, the effective operation of wave plates in the top and bottom modes can be described as first transforming $|VT\rangle \rightarrow \mu _T|HT\rangle + \nu _T|VT\rangle$ and $|HB\rangle \rightarrow \mu _B|HB\rangle + \nu _B|VB\rangle .$ The parameters are set as $\mu _T = \sqrt{2}c^3(x_n),$ $\nu _T = \sqrt{2}{s^3(x_n)},$ $\mu _B = \sqrt{6}c^2(x_n)s(x_n),$ $\nu _B = \sqrt{6}c(x_n)s^2(x_n).$

This whole operation is unitary and can be described as $U(x)|HH\rangle = |\varphi (x)\rangle .$ The complex conjugate of operation *U*(*x*) is $U^\dagger (x')$ and it can be used to express the kernel as $\kappa (x',x) = |\langle HH|U^\dagger (x')U(x)|HH\rangle |^2.$ Thus, the circuit $U^\dagger (x')$ for projecting the state $|\varphi (x)\rangle$ to $|\varphi (x')\rangle$ can be constructed as the inverse of the feature embedding *U*(*x*) circuit, but for setup parameters set for $x'$. The next action of the plates in the top and bottom rails is to perform a reverse transformation, but for $x_n=x'_n.$ Next, the plates flip the polarizations in the respective rails. Now, the interesting part of the engineered state is in the top rail with flipped polarization. To implement $U(x')^\dagger ,$ the last pair of waveplates is used both to flip the polarization and to perform the Hadamard transformation. Finally, the *PBS* transmits only *H*-polarized photons for further processing.

The procedure of measuring the kernel $\kappa (x',x)$ can be extended to include additional dimensions, resulting in measuring the kernel ${{\bar{\kappa }}}(x',x)=\kappa (x',x)\cos ^2(y-y')$ following from FM (11). Instead of the transformation $U^\dagger (x')U(x),$ we consider $R^\dagger (y')U^\dagger (x')U(x)R(y),$ where $R(y)= e^{2iy}|H\rangle \langle H|$ is a phase shift applied to a preselected *H*-polarized photon in the bottom part of the setup, and $R^\dagger (y')= e^{-2iy'}|H\rangle \langle H|$ is a phase shift to the postselected *H*-polarized photon in the same part of the setup. The phase difference between the postselected upper and lower *H*-polarized photons can be measured as $\cos ^2(y-y').$ This is done with $PBS'$ which transmits diagonally-polarized photons $|D\rangle = (|H\rangle +|V\rangle )/\sqrt{2}$ and reflects antidiagonal photons $|A\rangle = (|H\rangle -|V\rangle )/\sqrt{2},$ and polarization-resolving single-photon detectors (see caption of Fig. 3).
